# Altered Whole-Brain Functional Topological Organization and Cognitive Function in Type 2 Diabetes Mellitus Patients

**DOI:** 10.3389/fneur.2019.00599

**Published:** 2019-06-05

**Authors:** Chunhong Qin, Yi Liang, Xin Tan, Xi Leng, Huan Lin, Hui Zeng, Chi Zhang, Jinquan Yang, Yifan Li, Yanting Zheng, Shijun Qiu

**Affiliations:** ^1^Department of Radiology, First Affiliated Hospital of Guangzhou University of Chinese Medicine, Guangzhou, China; ^2^Department of Radiology, Zhujiang Hospital of Southern Medical University, Guangzhou, China; ^3^Guangzhou University of Chinese Medicine, Guangzhou, China

**Keywords:** type 2 diabetes mellitus, cognitive function, resting-state functional magnetic resonance imaging, topological organization, graph theoretical analysis

## Abstract

Type 2 diabetes mellitus (T2DM) is associated with cognitive dysfunction and may even progress to dementia. However, the underlying mechanism of altered functional topological organization and cognitive impairments remains unclear. This study explored the topological properties of functional whole brain networks in T2DM patients with graph theoretical analysis using a resting-state functional magnetic resonance imaging (rs-fMRI) technique. Thirty T2DM patients (aged 51.77 ± 1.42 years) and 30 sex-, age-, and education-matched healthy controls (HCs) (aged 48.87 ± 0.98 years) underwent resting-state functional imaging in a 3.0 T MR scanner in addition to detailed neuropsychological and laboratory tests. Then, graph theoretical network analysis was performed to explore the global and nodal topological alterations in the functional whole brain networks of the T2DM patients. Finally, correlation analyses were performed to investigate the relationship between the altered topological parameters, cognitive performances and clinical variables. Compared to HCs, we found that T2DM patients displayed worse performances in general cognitive function and several cognitive domains, including episodic memory, attention and executive function. In addition, T2DM patients showed a higher small-worldness (σ), a higher normalized clustering coefficient (γ) and a higher local efficiency (E_loc_). Moreover, decreased nodal topological properties were mainly distributed in the occipital lobes, frontal lobes, left median cingulate and paracingulate gyri, and left amygdala, while increased nodal topological properties were mainly distributed in the right gyrus rectus, right anterior cingulate and paracingulate gyri, right posterior cingulate gyrus, bilateral caudate nucleus, bilateral cerebellum 3, bilateral cerebellum crus 1, vermis (1, 2) and vermis 3. Some disrupted nodal topological properties were correlated with cognitive performance and HbA1c levels in T2DM patients. This study shows altered functional topological organization in T2DM patients, mainly suggesting a compensation mechanism of the functional whole brain network in the relatively early stage to counteract cognitive impairments.

## Introduction

With the growth of the aging population and changes in people's living habits, the prevalence of diabetes has been increasing year by year worldwide (1, 2). Type 2 diabetes mellitus (T2DM) is the most common type, accounting for more than 90% of all diabetes. Multiple studies have shown that T2DM can increase the risk of cognitive dysfunction and may even progress to dementia, including vascular dementia and Alzheimer's disease (AD) (3–5). However, the underlying mechanism of T2DM-induced cognitive dysfunction is still unclear.

Resting-state functional magnetic resonance imaging (rs-fMRI) has become an important neuroimaging research method to understand the neurophysiological mechanisms of T2DM-induced cognitive dysfunction. Recently, many studies have focused on the functional changes of T2DM patients in a resting state. Some previous studies reported altered regional homogeneity (ReHo) values or amplitude low-frequency fluctuations (ALFF) values in the occipital lobes, temporal lobes, frontal lobes, cingulate gyrus, and cerebellum (6–8). And functional connectivity (FC) measures the similarity of the time series of two relatively remote brain regions (9). Previous studies have mainly shown impaired FC in the default mode network (DMN), ventral attention network (VAN), and dorsal attention network (DAN) (10–13). Besides, these disrupted regional brain activity and FC were associated with multiple cognitive impairments in T2DM patients, including visual processing, memory, attention, and executive function. Specifically, these methods focused only on local spontaneous brain activity using ReHo and ALFF values or concentrated their investigations within specific brain networks using seed-based approaches or independent component analysis (ICA). However, T2DM-related abnormal brain areas are extensively distributed, and cognitive dysfunction involves comprehensive interactions between different brain areas. In this context, building the functional whole brain network is necessary to comprehensively understand the underlying mechanisms of T2DM-related cognitive impairments.

The human brain is a complex network that works in a small-world network model efficiently and optimally (14). Graph theoretical analysis can effectively reflect altered topological properties of complex human brain networks. In recent years, this method has been widely used in studies of various neuropsychiatric diseases, such as AD, epilepsy and schizophrenia (15–17), while T2DM-related research was rarely reported. In the studies of functional brain network, Chen et al. (18) found that the global topological properties of the T2DM patients without cognitive dysfunction were lower than those of HCs, however, van Bussel et al. (19) revealed that the global topological properties of the T2DM patient group and the prediabetic patient group were significantly higher than those of the healthy control group and associated with lower processing speed. It can be summarized that the definite alterations of topological properties existing in the functional brain networks of T2DM patients remain unclear and the relationship between altered topological property and cognitive function is unknown. In addition, the currently studies focused only on the cerebrum networks, however, the structure and function of the cerebellum was changed in T2DM patients (7, 20). Thence, we also need to include the cerebellum to comprehensively explore the topological properties of the whole brain network.

Therefore, in the present study, we used rs-fMRI with graph theoretical analysis to explore the characteristic changes of functional whole brain network topological properties in T2DM patients. We also analyzed the correlation among the altered topological parameters, cognitive performance and related clinical variables. We hope to provide some potential imaging biomarkers of T2DM-related cognitive deficits.

## Materials and Methods

### Participants

A total of 34 right-handed T2DM patients and 33 sex-, age-, and education-matched healthy controls (HCs) were recruited from the First Affiliated Hospital of Guangzhou University of Chinese Medicine. All participants received a detailed medical history interview and neurological examination. Clinical and demographic information were collected for all subjects, including biological test, blood pressure, body mass indicator (BMI), education level, alcohol consumption, smoking status, and duration of the disease (for T2DM patients only). The inclusion criteria were as follows: (1) All participants were between the ages of 40 and 65 years; (2) A standardized diagnosis of T2DM was confirmed based on medical history, medications used, fasting plasma glucose (FPG) levels (≥7.0 mmol/L) or 2-h OGTT glucose levels (≥11.1 mmol/L), which was in accordance with the diagnostic and classification criteria published by the American Diabetes Association (ADA) in 2014 (21); (3) HCs with FPG levels ≤ 6.1 mmol/L were included to this study. The exclusion criteria were as follows: (1) with clinically obvious complications, for example, the third or higher stages of diabetic retinopathy (based on the International Clinical Disease Severity Scale for diabetic retinopathy) (22), accompanying abnormal urinary microalbumin of nephropathy and with symptoms of peripheral neuropathy; (2)any history of severe hypoglycemia; (3) impaired glucose tolerance or impaired fasting glucose; (4) hypertension; (5) history of brain lesions such as tumor or stroke; (6) unrelated psychiatric or neurological disorder(s); (7) history of alcohol, smoke or drug abuse; (8) systemic diseases such as severe anemia, thyroid dysfunction, or acquired immune deficiency syndrome; and (9) MRI contraindications. This study was approved by the ethics committee of First Affiliated Hospital of Guangzhou University of Chinese Medicine. The current study was carried out in accordance with the principles of the Declaration of Helsinki and the approved guidelines. All participants signed informed consent before participating in the study.

### Neuropsychological Test

All participants completed detailed standardized cognitive assessment, which covered multiple cognitive domains. General cognitive function was assessed by the Chinese version of the Montréal Cognitive Assessment Scale-B (MoCA-B). Episodic verbal memory was measured by the Auditory Verbal Learning Test (AVLT). Working memory was evaluated by the Digit Span Test (DST). Attention was assessed by the Trail Making Test-A (TMT-A). Executive function was measured by the Grooved Pegboard Test. Spatial processing ability was evaluated by the Clock Drawing Test (CDT). It took approximately 40 min to finish all the tests.

### Image Acquisition

For each participant, whole-brain MRI data were acquired using a 3T scanner (Signa HDxt GE Medical Systems, USA) with an 8-channel head coil. The scan time was within 1 week after medical history interview, neurological examination and biological tests, and the same day after neuropsychological tests. First, all participants underwent routine whole-brain axial T1WI (TR/TE = 2,500/24 ms), T2WI (TR/TE = 5,100/130 ms), and T2 FLAIR (TR/TE = 9,000/120 ms) to rule out intracranial organic diseases, e.g., infarction, malformation, and tumor. Resting-state fMRI data were collected using a gradient-echo EPI sequence sensitive to blood oxygen level-dependent contrast with the following parameters: TR = 2,000 ms, TE = 30 ms, flip angle = 90°, thickness = 3 mm, gap = 1 mm, FOV = 220 × 220 mm, matrix = 64 × 64, slices = 36, 185 volumes. Sagittal high-resolution T1WI whole-brain images were acquired using 3D FSPGR sequences (TR = 8.15 ms, TE = 3.17 ms, Prep Time = 450 ms, flip angle = 12°, slice thickness = 1 mm, no gap, NEX = 1, FOV = 256 × 256 mm, matrix = 256 × 256, 188 sagittal slices). Earplugs and foam pads were used to reduce equipment noise and head motion during scanning. All participants were told to lay quietly in the scanner with their eyes closed, avoiding strong ideological activities but keeping awake.

Cerebral small vascular disease, mainly including white matter hyperintensities (WMHs) and lacunar infarction, may have an impact on brain function and cognitive function (23). In this study, these changes were assessed on T2WI and T2 FLAIR images according to the age-related white matter changes (ARWMC) scale (24). Two experienced radiologists who are blinded to group status separately performed the ratings and then reached a consensus through discussion. All participants with lacunar infarcts or a rating score > 2 were excluded. Consequently, 2 T2DM patients and 1 healthy control subject were excluded from this study.

### Image Preprocessing

Preprocessing of rs-fMRI data was performed with the Data Processing Assistant for rs-fMRI (DPARSF) (25) and Statistical Parametric Mapping (SPM8, http://www.fil.ion.ucl.ac.uk/spm) in the MATLAB version 2012a (MathWorks, Natick, MA, USA) platform. The first 10 time-points of the rs-fMRI images were removed to avoid the heterogeneity of the initial MRI signal. The 175 remaining volumes were preprocessed with the following steps: (1) slice timing, scanned image is a interleaved scan, so the slice order is [2:2:36 1:2:35] and the reference slice is 36; (2) realignment; two T2DM patients and 2 HCs were excluded from the study due to obvious head motion larger than 1.5 mm in any direction of x, y and z or 1.5° of any angular motion; (3) normalization, with the functional images coregistered to the high-resolution T1WI images; subsequently, the coregistered images were normalized into a 3 × 3 × 3 mm^3^ Montreal Neurological Institute (MNI) 152 template; (4) smoothed with a 6 mm full-width half-maximum isotropic Gaussian kernel; (5) linear detrending and temporal filtering at the 0.01~0.08 Hz band; (6) certain variables were regressed out: nuisance covariates including the white matter (WM) and the cerebrospinal fluid (CSF) signals as well as the 24 motion parameters (26); but the mean global signal was not regressed out from the data (27).

### Functional Whole Brain Network Construction and Graph Analysis

#### Node and Edge Definitions

Network construction and analysis were performed with the GRETNA package (http://www.nitrc.org/projects/gretna), a graph theoretical network analysis toolbox for imaging connectomics. In the present study, with the automated anatomical labeling (AAL) atlas, including the cerebellum, we constructed the whole brain functional networks of all participants. First, the whole brain was parcellated into 116 different brain regions, containing 90 cerebrum areas and 26 cerebellum areas, representing the nodes of the network. Second, the functional connectivity between each pair of segmented brain regions was calculated using the Pearson correlation coefficients, representing the edges of the network. Third, a whole brain 116 × 116 correlation matrix was constructed for each participant and then translated into binarized matrices. Finally, a Fisher's r-to-z transformation was conducted to convert the individual correlation maps into z-scored maps to promote normality.

#### Threshold Selection and Network Analysis

In this study, whole brain functional networks were constructed based on an undirected and unweighted method. For all participants, the brain functional networks should be thresholded by a sparsity value to ensure that all resultant networks have the same number of edges and that the number of spurious edges is minimized (28, 29). However, no golden criteria are available for which sparsity value is currently the most biologically meaningful. According to previous studies (30, 31), a sparsity range of 0.05–0.5 with an interval of 0.01 was chosen, and the remaining fraction of edges was calculated in the functional network for each participant. For each sparsity threshold, eight global and nodal network parameters were computed. The global network measures included five parameters (the normalized clustering coefficient γ, the normalized characteristic path length λ, the small-worldness σ, local efficiency E_loc_ and global efficiency E_glob_). The nodal network measures included three parameters (nodal degree, nodal efficiency and nodal betweenness). In addition, for each participant, to assess whether the network had small-world property, the network measures were normalized to comparable values from random networks (*N* = 100). Furthermore, the area under curves (AUCs), which are sensitive at detecting topological alterations of brain disorders (32), were calculated for each parameter over the entire sparsity range (0.05 ≤ Sp ≤ 0.5).

### Statistical Analysis

The demographic and clinical characteristics plus neuropsychological assessment of the T2DM patients and HCs were analyzed using the IBM Statistical Package for the Social Sciences 20.0 software (IBM SPSS Inc., Chicago, IL, USA). For continuous variables, independent two-sample *t*-tests or Mann-Whitney non-parametric tests were used, according to whether they met the normal distribution and variance homogeneity. The chi-square test was used to evaluate the differences in results between the genders within the groups. With gender, age, education years, and BMI as covariates, the between-group differences in the global parameters (γ, λ, σ, E_loc_, and E_glob_), nodal parameters (nodal degree, nodal efficiency, and nodal betweenness) and the AUC of each parameter were compared using two-sample *t*-tests (P, 0.05) over the entire sparsity range (0.05 ≤ Sp ≤ 0.5). The Bonferroni method was applied at a *p*-value of 0.05 to correct for multiple comparisons. In addition, with the same indicators as covariates, the correlation between the altered functional network topological parameters and neuropsychological tests and clinical variables were analyzed using partial correlation analysis. *P* < 0.05 was considered statistically significant.

## Results

### Clinical and Neuropsychological Results

Four T2DM patients and 3 HCs with obvious head motion or ARWMC scale rating scores > 2 were excluded, and 30 T2DM patients and 30 HCs were eventually included in the present study. The clinical and neuropsychological results of the T2DM patients and HCs are summarized in Table 1. The two groups were matched on age, sex, and education, and the BMI and blood lipid level were similar (*p* > 0.05), but both systolic blood pressure (SBP) (*p* = 0.013) and diastolic blood pressure (DBP) (*p* = 0.035) were higher in the T2DM patients. Compared with the HCs, the T2DM patients scored poorer on the MoCA-B (*p* = 0.010) and AVLT immediate recall tests (*p* = 0.016), spent much more time on the TMT-A (*p* = 0.018) and Grooved Pegboard Tests (p_R_ = 0.009, p_L_ = 0.025) and had no significant decreases in the other neuropsychological tests (*p* > 0.05).

**Table 1:** Clinical and neuropsychological results of T2DM patients and HCs.

	**T2DM patients (*n* = 30)**	**HCs (*n* = 30)**	***P*-value**
**Clinical characteristics**			
Age (years)	51.77 ± 1.42	48.87 ± 0.98	0.099
Sex (M/F)	18/12	18/12	1.000
Education (years)	10.70 ± 0.69	10.23 ± 0.61	0.614
BMI (kg/m^2^)	24.82 ± 0.56	24.18 ± 0.52	0.409
SBP (mmHg)	127.20 ± 2.35	120.03 ± 1.51	0.013^*^
DBP (mmHg)	82.80 ± 1.67	78.70 ± 0.88	0.035^*^
Total cholesterol mmol/L	4.71 ± 1.78	4.27 ± 0.96	0.240
Triglyceride (mmol/L)	1.54 ± 0.92	1.48 ± 0.50	0.755
LDL cholesterol (mmol/L)	3.34 ± 1.19	2.93 ± 0.4	0.084
HDL cholesterol (mmol/L)	1.07 ± 0.29	1.15 ± 0.46	0.424
**Alcohol consumption (%)**			
None/Low/High	83.3/16.7/0	90.0/10.0/0	–
**Smoking status (%)**			
Never/Former/Current	80.0/13.3/6.7	86.7/10.0/3.3	–
Duration of diabetes (years)	5.04 ± 4.46	–	–
Fasting blood glucose (mmol/L)	8.62 ± 3.44	5.03 ± 0.48	<0.001^*^
2h OGTT glucose (mmol/L)	18.53 ± 5.46	–	–
HbA1C (%)	8.54 ± 2.09	–	–
**Type 2 diabetes medication, yes (%)**			
Oral medication	50.0	–	–
Insulin medication	16.7	–	–
Insulin and oral medication	20.0	–	–
None(newly diagnosed)	13.3	–	–
**Cognitive scores**			
MoCA-B	25.23 ± 0.66	27.23 ± 0.34	0.010^*^
AVLT immediate recall	18.00 ± 0.80	21.17 ± 0.99	0.016^*^
AVLT short-term recall (5 min)	7.03 ± 0.43	7.97 ± 0.38	0.108
AVLT long-term delayed recall (20 min)	7.60 ± 0.52	7.70 ± 0.40	0.839
AVLT recognition	10.23 ± 0.43	11.00 ± 0.27	0.139
TMT-A	67.17 ± 5.94	50.57 ± 3.19	0.018^*^
Grooved Pegboard (R)	92.07 ± 5.63	75.30 ± 2.31	0.009^*^
Grooved Pegboard (L)	96.83 ± 5.30	83.57 ± 2.05	0.025^*^
DST	11.87 ± 0.39	12.73 ± 0.46	0.154
CDT	2.63 ± 0.11	2.77 ± 0.08	0.335

*Data are mean ± SD. BMI, body mass index; SBP, systolic blood pressure; DBP, diastolic blood pressure; MoCA-B, Montreal cognitive assessment-B; AVLT, Auditory verbal learning test; TMT-A, Trail making test-A; DST, digit span test; CDT, Clock drawing test*.

* *P < 0.05, which was considered statistically significant*.

### Small-World Properties of Resting-State Functional Networks

**Figure 1:**
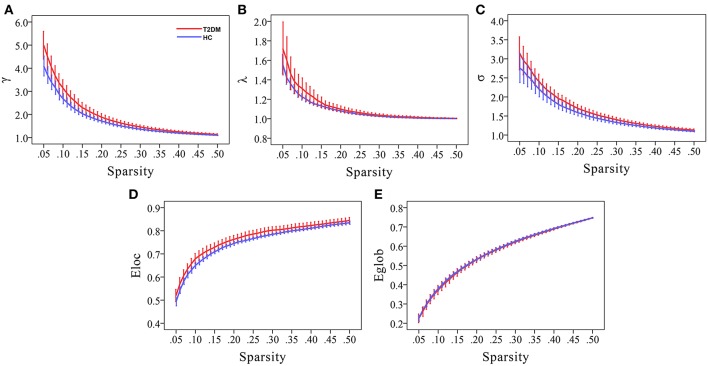
Small-world property and network efficiency measures of the whole brain network over the defined wide range of sparsity values of T2DM patients and healthy controls. Compared to random networks, graphs display that both the two groups had relatively higher normalized clustering coefficients (γ > 1), similar normalized characteristic path lengths (λ ≈ 1), and small-worldness σ (σ = γ/λ) > 1, that is, demonstrated small-world property **(A–C)**. Moreover, T2DM patients had higher local efficiency and similar global efficiency than HCs **(D,E)**.

Compared to random networks, the functional brain networks of the two groups had relatively higher normalized clustering coefficients (γ > 1), similar characteristic path lengths (λ ≈ 1), and small-worldness σ (σ = γ/λ) > 1, that is, demonstrated small-world property (Figures 1A–C).

### Altered Small-World Property and Network Efficiency in T2DM Patients

**Figure 2:**
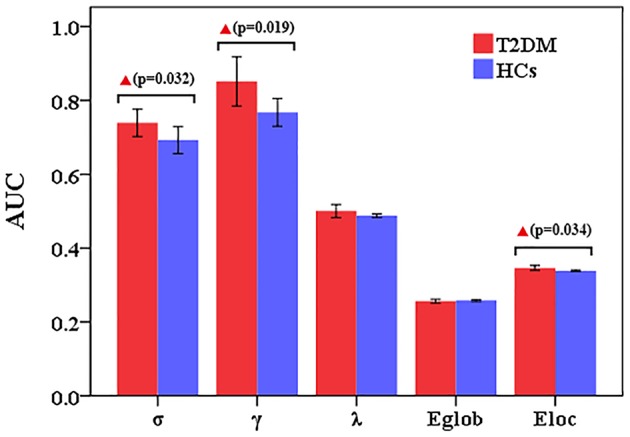
Altered small-world property and network efficiency measures of the whole brain network over the defined wide range of sparsity values between T2DM patients and healthy controls. Compared to HCs, T2DM patients showed the AUC values of γ (*p* = 0.019), σ (*p* = 0.032), and Eloc (*p* = 0.034) were significantly higher than HCs. However, λ and Eglob values were similar between T2DM patients and HCs.

Compared to HCs, T2DM patients showed increased γ values over the entire sparsity range (0.05 ≤ Sp ≤ 0.5), increased σ and E_loc_ values for a range of sparsity values (σ: 0.13 ≤ Sp ≤ 0.5 and E_loc:_ 0.09 ≤ Sp ≤ 0.31) (Figures 1A,C,D). Moreover, T2DM patients showed the AUC values of γ (*p* = 0.019), σ (*p* = 0.032), and E_loc_ (*p* = 0.034) were significantly higher than HCs (Figure 2). However, λ and E_glob_ values were similar between T2DM patients and HCs (*p* > 0.05) (Figures 1B,E, 2).

### Altered Nodal Topological Metrics in T2DM Patients

We identified 26 brain regions with altered nodal parameters between the T2DM patients and the HCs in at least one of the three nodal characteristics, which are reported in Table 2. Compared to the HCs, the T2DM patients showed decreased nodal parameters in frontal lobes [right precentral gyrus (PreCG.R), right supplementary motor area (SMA.R), left superior frontal gyrus, dorsolateral (SFGdor.L), left superior frontal gyrus, medial (SFGmed.L)], occipital lobes [right cuneus (CUN.R), bilateral lingual gyrus (LING.L&R), bilateral superior occipital gyrus (SOG.L&R) and middle occipital gyrus (MOG.L&R)], left amygdala (AMYG.L), left median cingulate and paracingulate gyri (DCG.L), and left supramarginal gyrus (SMG.L). However, T2DM patients exhibited increased nodal parameters in the right gyrus rectus (REC.R), right anterior cingulate and paracingulate gyri (ACG.R), right posterior cingulate gyrus (PCG.R), left angular gyrus (ANG.L), bilateral caudate nucleus (CAU.L&R), bilateral cerebellum 3 (CRBL3.L&R), bilateral cerebellum crus 1 (CRBLCrus1.L&R), vermis (1, 2) and vermis 3 compared to HCs. Most (14/26) brain regions demonstrated decreased nodal parameters in T2DM patients, with the remaining 12 brain regions showing increased nodal parameters.

**Table 2:** Brain regions with altered nodal parameters in T2DM patients.

**AAL no**.	**Brain Regions**		***p*****-values**		
	**Regions**	**Abbreviation**	**Nodal degree**	**Nodal efficiency**	**Nodal betweenness**
**T2DM <HC(14/26)**					
2	Right precentral gyrus	PreCG.R	0.052	**0.034**	**0.036**
3	Left superior frontal gyrus (dorsolateral)	SFGdor.L	0.519	0.196	**0.005**
20	Right supplementary motor area	SMA.R	0.141	0.156	**0.044**
23	Left superior frontal gyrus (medial)	SFGmed.L	0.335	0.183	**0.012**
33	Left median cingulate and paracingulate gyri	DCG.L	0.059	**0.037**	0.652
41	Left amygdala	AMYG.L	0.499	0.385	**0.026**
46	Right cuneus	CUN.R	0.054	**0.039**	0.652
47	Left lingual gyrus	LING.L	**0.008**	**0.009**	0.340
48	Right lingual gyrus	LING.R	**0.008**	**0.012**	0.531
49	Left superior occipital gyrus	SOG.L	**0.004**	**0.010**	0.135
50	Right superior occipital gyrus	SOG.R	**0.016**	**0.017**	0.656
51	Left middle occipital gyrus	MOG.L	0.058	**0.045**	**0.010**
52	Right middle occipital gyrus	MOG.R	**0.039**	**0.015**	**0.047**
63	Left supramarginal gyrus	SMG.L	0.058	**0.037**	**0.024**
**T2DM>HC(12/26)**					
28	Right gyrus rectus	REC.R	**0.048**	**0.034**	0.252
32	Right anterior cingulate and paracingulate gyri	ACG.R	0.930	0.597	**0.040**
36	Right posterior cingulate gyrus	PCG.R	0.488	0.903	**0.033**
65	Left angular gyrus	ANG.L	0.865	0.552	**0.040**
71	Left caudate nucleus	CAU.L	**0.001**	**0.004**	**0.005**
72	Right caudate nucleus	CAU.R	**0.030**	**0.030**	0.084
91	Left cerebellum crus1	CRBLCrus1.L	0.150	0.374	**0.049**
92	Right cerebellum crus1	CRBLCrus1.R	0.182	0.504	**0.025**
95	Left cerebellum 3	CRBL3.L	**0.010**	**0.011**	0.532
96	Right cerebellum 3	CRBL3.R	**0.004**	**0.001**	0.208
109	Vermis (1, 2)	Vermis(1, 2)	**0.015**	0.140	0.616
110	Vermis 3	Vermis 3	**0.003**	0.141	**0.023**

*AAL No., number of automated anatomical labeling. Note: Brain regions were considered abnormal in T2DM patients if they showed p < 0.05 compared to HCs in at least one of the three nodal parameters and boldface p-values were statistically significant*.

### Correlation Analyses Among Altered Network Parameters, Cognitive Function and Clinical Variables

**Figure 3:**
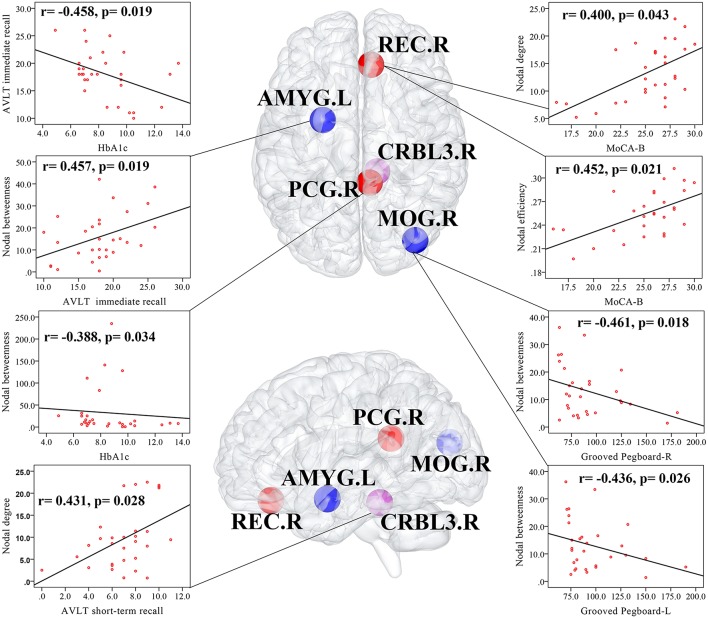
Correlations among altered network measures, cognitive function, and HbA1c in T2DM patients. Scatter plot displayed the relationship between the altered network parameters and clinical variables in T2DM patients. The blue ball represented the decreased nodal parameters and the red ball and purple ball represented the increased nodal parameters. MOG.R, right middle occipital gyrus; AMYG.L, left amygdala; REC.R, right gyrus rectus; PCG.R, right posterior cingulate gyrus; right cerebellum 3, CRBL3.R.

In T2DM patients, MoCA-B scores were positively correlated with the nodal degree (*r* = 0.400, *p* = 0.043) and nodal efficiency (*r* = 0.452, *p* = 0.021) of the REC.R. AVLT immediate recall scores were positively correlated with the nodal betweenness of the AMYG.L (*r* = 0.457, *p* = 0.019), and AVLT short-term recall scores were positively correlated with the nodal degree of the CRBL3.R (*r* = 0.431, *p* = 0.028). Both the Grooved Pegboard-R (*r* = −0.461, *p* = 0.018) and Grooved Pegboard-L (*r* = −0.436, *p* = 0.026) were negatively correlated with the nodal betweenness of the MOG.R. HbA1c was negatively correlated with the nodal betweenness of the PCG.R (*r* = −0.388, *p* = 0.034) and AVLT immediate recall scores (*r* = −0.458, *p* = 0.019). Correlation analyses were illustrated in Figure 3. No relationship was found in altered global network parameters.

## Discussion

In this cross-sectional study, we focused on the topological organization of rs-fMRI whole brain network of middle-aged T2DM patients without obvious complications using graph-based theoretical approaches. The results displayed that both the two groups exhibited small-world organization of their functional networks, but compared to HCs, T2DM patients showed (1) a higher normalized clustering coefficient (γ), a higher small-worldness (σ) and a higher local efficiency (E_loc_); (2) both decreased and increased nodal network parameters; (3) altered nodal network parameters of some brain regions were related to cognitive impairments and HbA1c. These findings provide new insight into the underlying functional neuropathological effects of T2DM-related cognitive impairments.

### Increased Global Network Measures

The small-world network, with a similar characteristic shortest path length and a higher clustering coefficient compared to the random network, is a highly integrated and optimized network model that can maximize efficiency and minimize information processing. Small-world networks were found not only in real-world networks, for example, social, traffic, and genetic networks, but also in functional, structural, and EEG human brain networks. In this study, the functional whole brain networks of both T2DM patients and HCs exhibited small-world organization, which was consistent with previous studies (18, 19, 32, 33).

As far as we know, normal human brain networks that combine a high σ, a high γ, and a high E_loc_ indicate a highly integrated and optimized network, a high local effectiveness in processing information and a high fault tolerance of the network. Our investigation showed this combination in T2DM patients, implying that the whole brain networks were better organized than those in HCs. The result seems unreasonable and converse because the networks of T2DM patients should be less, instead of better, organized. However, in the research of the rs-fMRI network among T2DM patients, prediabetes patients and healthy controls, van Bussel et al. (19) found results similar to our study. They held the view that before the appearance of clinically manifested cognitive decrements, the brain functional network may have already reorganized as a compensatory mechanism to counterwork the slight cognitive decrements. Once the functional reorganization fails, there will be a disrupted functional network and clinically manifested cognitive decrements will be discovered in T2DM patients. MoCA-B is widely used to assess general cognitive function and is more sensitive than MMSE. The mean MoCA-B score was 25.23 ± 0.66 in the T2DM patients in our study, which was slightly lower than the normal score 26, suggesting a stage of slight cognitive decrements. Besides, the included T2DM patients are middle-aged, with a short diabetes duration and well-controlled glucose levels, and without obviously complications, thus they may be relatively “healthy” patients. Therefore, the better organized whole brain networks in the T2DM patients of our study also supported the compensatory mechanism put forward by van Bussel et al. However, Chen et al. (18) showed longer path length and lower global efficiency but similar clustering coefficient and local efficiency in T2DM patients without mild cognitive impairment (MCI), indicating a less rather than better organized functional network, which is not consistent with our findings. These differences may be attributed to the severity of the disease condition or the sensitivity of the different neuropsychological tests and need to be discussed through longitudinal, large sample, long-term investigations in the future. Moreover, disrupted structural networks have already been reported via graph theoretical network analysis in T2DM patients (32, 33), but the relationship between the brain structural network and the functional network is still unknown. We believe that it is meaningful to combine structural networks with functional networks using theoretical network analysis to explore the underlying mechanism of T2DM-related cognitive function in the future.

### Decreased Nodal Network Measures

Nodal network parameters (nodal degree, nodal efficiency and nodal betweenness) can detect the activity, importance and influence of a region in network communication. In our study, reduced nodal parameters were observed in the occipital lobes, frontal lobes, left median cingulate and paracingulate gyri, left amygdalaleft and supramarginal gyrus. And the decreased nodal parameters of occipital lobes existed in CUN.R, bilateral LING, SOG, and MOG. Recently, a study found that the degree centrality of the LING was significantly reduced in T2DM patients and the connectivity within the LING-related visual network was diffusely decreased (34). Moreover, they found positive correlations of the occipital connectivity with visual memory and executive performance. In addition, in the earlier studies, T2DM patients showed not only decreased volume and brain metabolites (35, 36) but also decreased ReHo and ALFF values of the occipital lobes, especially in CUN, LING, SOG, MOG and calcarine gyrus (CAL) (6, 8, 37). In our study, the nodal degree of the MOG.R was negatively correlated with the consumed time of the Grooved Pegboard Test (a scale reflecting execution function), suggesting that a decreased nodal degree of the MOG.R may be attributed to reduced performance in executive function.

The cingulate gyrus is the core node in the DMN and acts as a transportation hub during information transmission processing and participates in various cognitive functions. To the best of our knowledge, only one study showed that the increased degree centrality of the dorsal anterior cingulate cortex (dACC) and the increased connectivity of the dACC was related to higher FPG levels and better TMT-B performance in T2DM patients (34). But impaired functional activity of DMN has been widely reported in previous studies using ReHo, ALFF, seed-based approaches or independent component analysis (12, 38, 39). Our study found that the nodal parameters in the DCG.L and SMG.L were reduced, while the nodal parameters in the ACG.R and PCG.R were increased. These findings may be interpreted as the fact that the left hemisphere of the recruited right-handed participants are more active than the right hemisphere and more sensitive to pathological changes caused by hyperglycemia, thus, compensatory increases of the right cingulate gyrus will be made to maintain the brain function activities of the whole brain. Furthermore, the nodal betweenness of the PCG.R was negatively correlated with HbA1c, suggesting that controlling and monitoring the HbA1c value is of great significance for the development of diabetic encephalopathy.

The frontal lobe is the latest and most advanced part of brain development. It is widely accepted that the frontal lobes, especially prefrontal lobes, are primarily responsible for high-order cognitive control (40, 41), and appear to be vulnerable regions in T2DM patients by using functional connectivity and graph theoretical network analysis (13, 19, 32). In this study, as shown in Table 2, several frontal lobes (PreCG.R, SMA.R, SFGdor.L, and SFGmed.L) showed decreased nodal parameters, while the REC.R showed increased nodal parameters. In addition, increased nodal degree and nodal efficiency of the REC.R were related to higher MoCA-B scores. These results suggest that disrupted frontal topological properties may further explain the damaged neural mechanism and declined cognitive function in T2DM patients. The AMYG is located in the medial temporal lobe and is mainly involved in mood and memory. The AMYG.L performed decreased nodal betweenness and was related to worse performance in the AVLT immediate recall test, suggesting that its ability to participate in network information transmission was reduced and may partially explain the reason for memory loss in T2DM patients. Recently, Xia et al. (42) reported that T2DM patients may be accompanied by depressive mood, and depressed T2DM patients showed decreased AMYG FC when compared to non-depressed T2DM patients. However, our study did not assess depression-related scales, and this needs to be further discussed in future studies.

### Increased Nodal Network Measures

Finally, to the best of our knowledge, this study is the first to explore the topological properties of whole-brain (including cerebellum) functional networks using graph theoretical analysis in T2DM patients. In the previous studies of resting state functional MRI, increased ReHo or ALFF values and functional connectivity of the cerebellum posterior lobe and cerebellum culmen were reported in T2DM patients (7, 8, 43). They hold the view that cerebellum, especially the cerebellum posterior lobe, may play a role of compensation. And this study demonstrated increased nodal parameters in the bilateral cerebellum 3, bilateral cerebellum crus 1, vermis (1, 2) and vermis 3, which was partly consistent with the previous studies. Moreover, in the previous studies of structural MRI, decreased FA values of vermis (44) and increased MD values of bilateral cerebellum anterior and posterior lobes (45) were reported, and some decreased connections in cerebellar and cerebro-cerebellar circuit were found (20). These studies displayed that the cerebellum was both damaged in function and structure, but there was no report about the definite relationship between cerebellum and cognitive function in T2DM patients. The cerebellum not only plays an important role in motor control and coordination but also relates to some advanced cognitive functions, such as language, emotional modulation, episodic and working memory (46–48). In the present study, the nodal degree of the right cerebellum 3 was positively correlated with the AVLT short-term delayed recall score, suggesting a close relationship between the cerebellum and memory. Therefore, we speculate that in the relatively early stage, the elevated brain functional activity of the cerebellum, especially the cerebellum posterior lobe, can recruit more nerve resources as a compensation mechanism to slow the process of cognitive decline. This may also explain why the local efficiency of T2DM patients is higher than that of HCs from another expect, which may be due to the compensation mechanism of the increased nodal properties in the these brain regions mentioned above.

## Limitations

This study had some limitations. First, it was a cross-sectional study that did not assess the progression of functional network changes and had a relatively small sample size. Second, the medication of T2DM patients was not completely identical, so medication confounding effects may exist. Therefore, the effect of medication needs to be investigated in future studies. Third, previous studies reported that T2DM patients may have depression, but our study did not assess the patient's mood state with a detailed depression scale. Moreover, according to the presence depression, we can divide these T2DM patients into different subgroups and further explore the differences between them. Finally, we only explored the relationship between the brain functional network and cognitive performance in T2DM. The incorporation of a structural network allowed us to examine whether the functional changes underlying cognitive dysfunction in T2DM are associated with structural network alterations. Further studies that combine multimodal imaging techniques will be helpful to interpret this issue.

## Conclusion

In summary, this study displayed disrupted functional networks in middle-aged T2DM patients with mild cognitive impairments, demonstrating a more efficient global topological organization and showing both decreased and increased nodal parameters. This may suggest a compensation mechanism for cognitive decline in terms of functional reorganization of the whole brain networks. Furthermore, the study demonstrated that graph theoretical network analysis provided novel insight and the results may serve as potential imaging biomarkers for subtle whole brain alterations of T2DM-related cognitive decline.

## Ethics Statement

This study was approved by the ethics committee of First Affiliated Hospital of Guangzhou University of Chinese Medicine. The current study was carried out in accordance with the principles of the Declaration of Helsinki and the approved guidelines. All participants signed informed consent before participating in the study.

## Author Contributions

CQ carried out the data collection, analysis and interpretation, and drafted the initial article. YL, XT, HZ, JY, YfL, and YZ participated in the data collection and interpretation. XL, HL, CZ and SQ contributed to the conception and design of the study, interpretation of data, and manuscript revision. All authors read the final manuscript and approved it for publication.

### Conflict of Interest Statement

The authors declare that the research was conducted in the absence of any commercial or financial relationships that could be construed as a potential conflict of interest.
